# A Review of Microsatellite Markers and Their Applications in Rice Breeding Programs to Improve Blast Disease Resistance

**DOI:** 10.3390/ijms141122499

**Published:** 2013-11-14

**Authors:** Gous Miah, Mohd Y. Rafii, Mohd R. Ismail, Adam B. Puteh, Harun A. Rahim, Kh. Nurul Islam, Mohammad Abdul Latif

**Affiliations:** 1 Laboratory of Food Crops, Institute of Tropical Agriculture, Universiti Putra Malaysia, 43400 UPM Serdang, Selangor, Malaysia; E-Mails: g_miah@yahoo.co.uk (G.M.); razi@upm.edu.my (M.R.I.); 2 Department of Crop Science, Faculty of Agriculture, Universiti Putra Malaysia, 43400 UPM Serdang, Selangor, Malaysia; E-Mails: adam@upm.edu.my (A.B.P.); alatif1965@yahoo.com (M.A.L.); 3 Agrotechnology and Bioscience Division, Malaysian Nuclear Agency, 43000 Kajang, Selangor, Malaysia; E-Mail: rahim@nuclearmalaysia.gov.my; 4 Laboratory of Anatomy and Histology, Department of Veterinary Preclinical Sciences, Faculty of Veterinary Medicine, Universiti Putra Malaysia, 43400 UPM Serdang, Selangor, Malaysia; E-Mail: kislam_2013@yahoo.com; 5 Bangladesh Rice Research Institute, Gazipur 1701, Bangladesh

**Keywords:** simple sequence repeats, marker development and application, blast resistance, marker assisted selection, rice breeding

## Abstract

Over the last few decades, the use of molecular markers has played an increasing role in rice breeding and genetics. Of the different types of molecular markers, microsatellites have been utilized most extensively, because they can be readily amplified by PCR and the large amount of allelic variation at each locus. Microsatellites are also known as simple sequence repeats (SSR), and they are typically composed of 1–6 nucleotide repeats. These markers are abundant, distributed throughout the genome and are highly polymorphic compared with other genetic markers, as well as being species-specific and co-dominant. For these reasons, they have become increasingly important genetic markers in rice breeding programs. The evolution of new biotypes of pests and diseases as well as the pressures of climate change pose serious challenges to rice breeders, who would like to increase rice production by introducing resistance to multiple biotic and abiotic stresses. Recent advances in rice genomics have now made it possible to identify and map a number of genes through linkage to existing DNA markers. Among the more noteworthy examples of genes that have been tightly linked to molecular markers in rice are those that confer resistance or tolerance to blast. Therefore, in combination with conventional breeding approaches, marker-assisted selection (MAS) can be used to monitor the presence or lack of these genes in breeding populations. For example, marker-assisted backcross breeding has been used to integrate important genes with significant biological effects into a number of commonly grown rice varieties. The use of cost-effective, finely mapped microsatellite markers and MAS strategies should provide opportunities for breeders to develop high-yield, blast resistance rice cultivars. The aim of this review is to summarize the current knowledge concerning the linkage of microsatellite markers to rice blast resistance genes, as well as to explore the use of MAS in rice breeding programs aimed at improving blast resistance in this species. We also discuss the various advantages, disadvantages and uses of microsatellite markers relative to other molecular marker types.

## 1. Introduction

Rice blast, which is caused by *Pyricularia grisea* (Cooke) Sacc., the anamorphous state of *Magnaporthe grisea* (T.T. Hebert) Barr [1], is the primary limiting biotic factor for rice production throughout the world. The use of resistant cultivars is the most effective and economical way to control rice blast disease, and therefore, breeding efforts to develop new resistant cultivars continue to be a priority for rice breeding programs. One of the challenges facing breeders during the development of improved rice cultivars, be they for conventional or organic agriculture, is the incorporation of disease resistance. Since the idea of indirect selection using genetic markers was first reported by Sax [2] over 80 years ago, and particularly in the last few decades, new technologies have emerged that allow breeders to more easily select changes at the DNA level. Much of the progress to date has centered on marker-assisted backcrossing or the pyramiding of genes against rice blast [3]. Presently, the integration of genomics and molecular-based breeding strategies for developing disease resistance, with gene-based marker assisted selection (MAS) being particularly effective, is a powerful method for efficient selection. In this context, pyramiding several major resistance genes into a valuable genetic background is simplified by the use of marker-based selection [4]. In short, the development and use of DNA markers has irrevocably changed the fields of rice genetics and breeding.

Molecular markers are now widely used to track loci and genome regions in crop breeding programs, as large numbers of molecular markers that are tightly linked to disease resistance traits are available in most major crop species [5–7]. The majority of molecular markers have been isolated from genomic DNA libraries or from libraries of randomly amplified PCR fragments. Molecular markers are essential for mapping genes of interest, marker-assisted breeding, and cloning genes using mapping-based cloning strategies [8]. Other uses of molecular markers include gene introgression through backcrossing, germplasm characterization and phylogenetic analysis [6]. Of the various classes of existing markers, microsatellites have emerged as the markers of choice for plant breeding applications [9]. Restriction fragment length polymorphism (RFLP) analyses are not easily scalable to high-throughput methods, and random amplification of polymorphic DNA (RAPD) assays are often not reproducible or transferable between laboratories. Although both microsatellites and amplified fragment length polymorphisms (AFLPs) can both be used to efficiently identify polymorphisms, microsatellite-based methods are more readily automated [10]. In addition, AFLP analysis is not always straightforward, as seemingly individual bands may actually be composed of multiple fragments [11], particularly when using large genomic templates. Furthermore, molecular markers can be used to estimate overall genetic variability, determine the proportion of a genome that has been introgressed from a donor, identify genes that are phenotypically related to a particular analyzed trait, and select for traits during multiple rounds of introgression [12].

Identifying resistance genes using molecular markers is the basic prerequisite for performing MAS in resistance breeding programs [13]. PCR-based microsatellite markers have attracted a great deal of attention due to several key advantages. Most importantly, they are hypervariable, abundant and well distributed throughout the rice genome. Furthermore, these markers are readily accessible through published linkage maps and public databases, and they permit the differentiation between homozygous and heterozygous individuals [14,15]. Overall, the speed, reliability and cost-effectiveness of acrylamide gel-based microsatellite analysis make this method an attractive tool for MAS in blast resistance breeding programs [16].

In recent years, microsatellite markers have been widely used to screen, characterize and evaluate genetic diversity in cereal species [17]. In particular, microsatellite-based methods offer an attractive high-throughput and non-labor-intensive way to tag blast resistance genes in breeding programs. A number of microsatellite markers have been developed from publicly available databases (http://www.gramene.org) that are tightly linked with the *Pi-ta*^2^, *Pi-k*^h^ and *Pi-b* resistance genes [18,19]. Furthermore, the development of molecular methods to efficiently identify novel resistance genes has the potential to greatly improve modern cultivars, and such methods would help accelerate the application of MAS and marker-assisted backcross (MAB) breeding in rice improvement programs. Use of these markers should also facilitate the development of multiline cultivars that carry one or more blast resistance genes, without the need for pathogenic inoculation or phenotyping. Microsatellite markers have been integrated into the molecular genetic maps of a number of plant species, and they have been successfully used to perform gene-mapping, population and evolutionary studies for the purpose of cultivar development. In this review, we summarize the known microsatellite markers that are linked to rice blast disease resistance. Furthermore, we investigate the organization of microsatellites within the rice genome and evaluate their usefulness as genetic markers. In particular, this review focuses on the availability of DNA markers linked to blast resistance in rice improvement programs and their potential use in MAS. The main objectives of this review are the following: (i) to help increase the efficiency of MAS in breeding-program crosses, thus improving the chances of developing new lines for commercial release; (ii) to review the basic principles and characteristics of commonly used microsatellite markers; (iii) to outline the advantages and limitations of these markers; and (iv) to provide examples of how microsatellites have been used in molecular breeding programs for blast resistance.

## 2. Microsatellites

The term microsatellite was first coined by Litt and Luty [20]. Microsatellites are simple repeated motifs consisting of 1 to 6 base pairs, and they can be found in both coding and non-coding regions. The mutation rate of this type of genetic marker has been estimated to be between 10^−2^ and 10^−4^ per generation. The primary advantage of microsatellites as genetic markers is that they are inherited in a Mendelian fashion as codominant markers. Furthermore, high polymorphism rates, high abundance and a broad distribution throughout the genome have made microsatellites one of the most popular genetic markers for use in plant breeding programs [21,22]. However, significant drawbacks do exist with respect to using microsatellite-based methods, including relatively high development costs and technical challenges during the construction of enriched libraries and species-specific primers.

## 3. Microsatellites: The Marker of Choice

The underlying principles and methodologies of various types of molecular markers that have been used in plants are shown in Table 1. The main challenge for researchers lies in selecting one or more of these markers for their specific purposes. The ideal type of genetic marker should be highly polymorphic, show codominant inheritance and be evenly distributed throughout the genome. In addition, particular marker sequences should be easy to access, and analyses should be low cost, high-throughput, reproducible, and transferable between laboratories, populations and/or species. Unfortunately, no marker type currently exists that meet all these requirements. However, based on the particular type of study, one can still choose among the different molecular marker systems to find the one that best suits your needs. A number of factors should be considered when choosing between the various molecular markers:

(a) Marker system availability
(b) Complexity of the technique and time investment
(c) Estimated polymorphism levels within the study population
(d) Quantity and quality of available DNA available
(e) Transferability between laboratories, populations, pedigrees and species
(f) The size and structure of the population to be studied
(g) Availability of skilled workers and equipment
(h) Cost per data-point and funding availability
(i) Method of marker inheritance (e.g., dominant *vs.* codominant) and the type of genetic information needed in the population [23–27].

In most areas of molecular genetics, microsatellites are now the marker of choice [28]. Microsatellites are also outstanding markers for fluorescent techniques, high-throughput analyses and multiplexing. However, high development costs and the significant effort required to design primer sets for a given study species remain limitations for using microsatellite markers to screen genomic libraries.

## 4. Classification of Microsatellites

Microsatellites can be classified based on size, the nature of the repeated unit or their position within the genome. With respect to the number of nucleotides per repeat unit, microsatellites can be classified as mono-, di-, tri-, tetra-, penta- or hexa-nucleotide repeats (Table 2). Depending upon the arrangement of nucleotides within the repeat motifs, Weber [34] used the terms perfect, imperfect and compound to classify microsatellites, whereas Wang *et al.* [35] coined the terms simple perfect, simple imperfect, compound perfect and compound imperfect. Perfect repeats are tandem arrays of a single repeat motif, whereas imperfect repeats consist of otherwise perfect repeats that are sometimes interrupted by non-repeat sequences. In compound microsatellites, two basic repeat motifs are present together in various configurations. Jarne and Lagoda [36] coined the terms pure and interrupted to describe perfect or imperfect repeats, respectively, as shown in Table 2.

## 5. Comparative Advantages and Disadvantages of Microsatellite Markers

The use of molecular markers is still prohibitively expensive for most large-scale applications in rice breeding programs. Therefore, MAS methods are currently used for more targeted applications [37]. The advantages and disadvantages of the most commonly used markers are presented in Table 3.

## 6. Utility of Microsatellite Markers

A significant advance in the practical utilization of molecular markers was the development of microsatellite markers [38]. Microsatellites are extremely informative markers that can be used for a variety of population genetics studies. Microsatellites are also considered ideal markers for genetic mapping studies [36,39,40] and germplasm evolution. For instance, Sakai *et al.* [41] used microsatellite markers to investigate the genomic evolution of the African cultivated rice *Oryza glaberrima* and the Asian cultivated rice *Oryza sativa*, and they identified 2451 microsatellite from these two genomes. Of these 2451 microsatellites, 883 were identical among the three studied genomes (*Oryza glaberrima*, *Oryza sativa indica* and *Oryza sativa japonica*), whereas the remaining 1568 markers were variable. Microsatellite markers show a higher degree of polymorphism in rice compared with restriction fragment length polymorphisms (RFLPs), and they are also suitable for evaluating genetic diversity among closely related rice cultivars [42]. For MAS applications, microsatellite markers with high polymorphism information content (PIC) values are generally more useful. Parida *et al.* [43] observed higher PIC in *indica* strains compared with aromatic, *japonica* and aus/wild strains, which agreed well with earlier observations using microsatellite and SNP markers [44–51]. Using their model, they were also able to identify four major genetically distinct groups within rice—*indica*, *japonica*, aromatics and aus/wild—based on population-structure analyses using microsatellites and SNP markers [45,48–50]. The estimated genetic diversity among the rice subpopulations in their study was significantly higher than previously observed using microsatellite and SNP markers [47,49] but was comparable to the diversity detected within a larger group of rice genotypes analyzed using microsatellite markers [51]. There exist several other examples of using microsatellites for these types of studies. Another interesting application of microsatellites in rice breeding was described by Liu and Wu [52]. In a study by Edwards *et al.* [53], genotyping microarrays utilizing single feature polymorphisms (SFPs) were used to assess the genetic variability across 20 diverse *O. sativa* varieties representing five different subpopulations, as determined using STRUCTURE [54] analysis with 169 microsatellite markers [49]. The highest level of polymorphism (66.2%) was found between the *temperate japonica* and *indica* subpopulations. The lowest levels of polymorphism were found within the *temperate japonica* sub-population (10.4%), which is also the least diverse subpopulation according to microsatellite markers [49].

**Figure 1:**
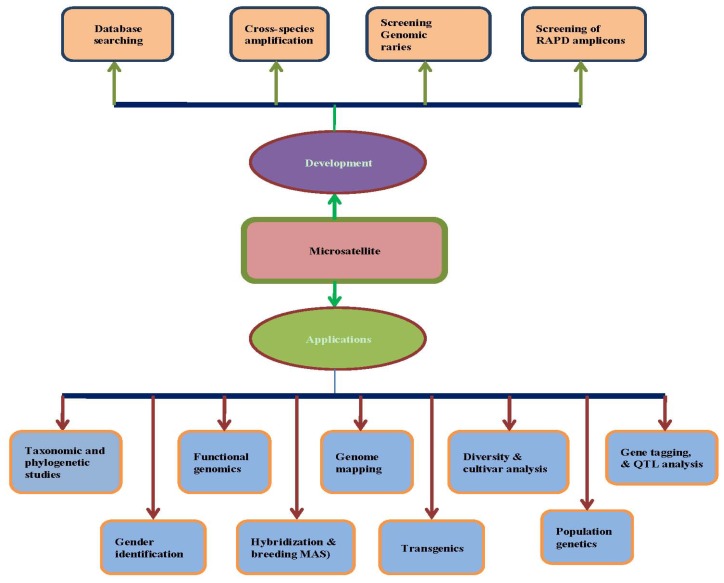
Development and applications of microsatellite markers at a glance [31].

As the order of markers along chromosomes is conserved within species and generally conserved between related species, this information can be used to create linkage maps, which are becoming increasingly available and detailed for a large number of species. This information is available for many marker types, and microsatellite-based maps are particularly well suited for genotyping [55]. For example, by analyzing such mapping information, it is possible to determine the fates of distinct parts of the genome over the course of developing multi-line varieties and composite-crosses [56]. Summaries of the various applications of microsatellite-based markers in plants are presented in Table 4 and Figure 1. However, it should be noted that each type of microsatellite-based marker possesses its own set of advantages and disadvantages based on mode of inheritance, informativeness, reproducibility, or procedural complexity, as well as economic concerns, such as cost and labor time. Therefore, the decision concerning which type of marker to use should be carefully considered based on the nature of the particular research project.

## 7. Microsatellite Marker Development

Completely sequenced genomes provide the basis upon which to design a large number of gene-based microsatellite markers. For example, rice (*Oryza sativa* L.) was the first cereal to have its genome completely sequenced, which has enabled the development of a large number of microsatellite markers [58]. Recently, Zhang *et al.* [59] developed 52,485 microsatellite markers that are polymorphic between *indica* and *japonica*. However, the difficulty now lies in choosing the most useful and informative microsatellite markers from such large datasets to use in rice genotyping applications. This problem can be overcome by constructing smaller, informative microsatellite marker databases composed of markers located in potentially functional genic sequences with relatively high polymorphic potential. Considering the excellent genetic attributes and higher predicted informativeness of genic non-coding microsatellite (GNMS) markers, Parida *et al.* [60] identified 19,555 perfect GNMS repeats on chromosomes 1 and 12 in rice. With the entire rice genome now sequenced, microsatellite markers can be developed within a few thousand base pairs of any gene. For example, a study by Goff *et al.* [61] suggests the presence, on average, of one microsatellite repeat (defined as at least eight repeats of a 2–4 bp motif) every 8 kb, yielding a total of 48,351 markers in the entire genome. Of course, not all these repeats can be developed into microsatellite markers [37], which can also be identified by screening database sequences or by screening libraries of clones (Figure 1).

PCR primers for amplifying microsatellite markers were designed by Rozen and Skaletsky [62] using the online program Primer3 by subjectively choosing primers flanking the repeat regions. The primers were then ordered from various commercial vendors and tested for their ability to amplify the microsatellites and differentiate polymorphisms among the parental lines used in the *Pi-z* mapping studies. Markers RM527 and RM6836, which were previously localized near the *Pi-z* locus [63], were obtained from the gramene website (http://www.gramene.org/) [64] and also included in primer testing [65].

Next-generation sequencing technologies (HighSSR, Roche 454 GS FLX) are now being used for microsatellite discovery with significant savings in cost and time [66–70]. Rapid progress in DNA sequencing technologies has substantially reduced costs while exponentially increasing throughput and accuracy. Currently, the most cost effective next-generation sequencing platform is the IlluminaHiSeq2000 [71], which can reduce costs 3400-fold relative to traditional sequencing methods; it is reasonable to expect that continued improvements will lead to even lower costs [72].

Fjellstrom *et al.* [73] designed DNA markers using four different methods. Five of these markers (RM101, RM138, RM144, RM155, and RM166) were based on an earlier set of microsatellites identified at Texas A & M University identified by screening the NCBI public DNA sequence database for repeated sequences, as described in Temnykh *et al.* [42]. The genomic locations of *Pi-b*, *Pi-k*, and *Pi-ta*^2^ and their actual linkages were confirmed by mapping these genes in several of the populations described by Conaway *et al.* [19]. By mapping these initial markers relative to microsatellite markers developed using traditional methods at Cornell University [42], three additional tightly linked markers were subsequently identified: RM208, RM224, and RM266. After identifying candidate microsatellite markers from public database resources, the authors mapped these markers near to the blast resistance genes *Pi-b*, *Pi-k*, and *Pi-ta*^2^ on rice chromosomes 2, 11 and 12, respectively. The public release of the Monsanto rice microsatellite database then allowed for the development of two additional linked microsatellite markers, which released as RM1233 and RM7102 [14]. Although DNA markers for rice blast resistance have been developed, most are not suitable for routine use in the MAS program involving large numbers of progeny. A dominant marker for the *Pi-b* gene, *Pibdom*, has also been developed based on the sequence of the cloned *Pi-b* gene [74] (GenBank accession AB013448). These markers should facilitate the introgression and pyramiding of these three blast resistance genes into new rice cultivars and elite lines [73]. In addition, a high-density microsatellite map with a genome coverage of approximately one microsatellite per 0.5 cM has been developed by the International Rice Microsatellite Initiative (IRMI) [75], which can be used for developing tightly linked markers for a variety agronomic traits, including blast resistance. Accessibility to the complete genomic sequences of the rice subspecies *indica* and *japonica* under public domain (http://rgp.dna.affrc.go.jp; http://www.genomics.org.cn) has enabled rice researchers to generate additional markers for the fine-scale mapping of targeted genes. For the purposes of obtaining a high-density linkage map for fine-scale mapping within their target region, new SSR, InDel, and CAPS markers were developed [76] using the publicly available rice genome sequence (http://rgp.dna.affrc.go.jp).

In species with large genomes, the conversion of microsatellite-containing sequences into useful markers can be difficult [77–80]. The recovery percentage of functional microsatellite primers in such genomes is usually low, which can be due to (i) the amplification of complex, weak or nonspecific patterns; (ii) lack of amplification; or (iii) non-polymorphic PCR products. Therefore, researchers often choose to use tri- and tetra-nucleotide repeat motifs, as opposed to di-nucleotide motifs, as these generally yield fewer “stutter bands” [40,81]. However, di-nucleotide motifs are more common than tri- or tetra-nucleotide motifs and are therefore easier to use in combinatorial screens. The wide variety of DNA markers discovered and developed in rice is shown in Table 5.

Although microsatellite marker are considered to be the most practical genetic markers, their application remains somewhat limited due to the time and effort needed to develop them. Two general strategies are used to identify and create microsatellite markers: (i) searching for sequences containing microsatellites within databases; and (ii) constructing and screening genomic libraries with probes complementary to microsatellite sequences. Two websites recommended by Romero *et al.* [82] are shown in Table 6.

## 8. Abundance of Microsatellite Motifs in Rice

The frequency of microsatellite motifs varies significantly among different organisms [39,83,84]. The most abundant microsatellite motif reported in plants is (AT)*n*, whereas (AC)*n* is most abundant motif in the human genome. Currently, there are no reliable estimates of the number of (AT)*n* or (GC)*n* sites in rice due to the difficulty of the hybridization-based screening methods used to detect these motifs [85,86]. The size of the rice genome is ~0.45 × 10^9^ bp [87], whereas the size of the human genome is ~6.6 times larger (3.0 × 10^9^ bp) [88]. These figures suggest that there should be one (AC)*n* site approximately every 360–450 kb in rice, compared with one every 40–80 kb in humans; similarly, it is estimated that there is one (GA)*n* motif every 225–330 kb in rice [85,86]. Three hundred and twenty three microsatellite markers identified by library screening [42,86,89,90] and GenBank searches of rice sequences [42,83,86] have been localized on the rice genetic map. The vast majority of these markers contain di- and tri-nucleotide motifs, with only seven loci containing tetra-nucleotide repeats, such as (AATT)*n*, (TTCC)*n*, (GATG)*n*, (ATGT)*n*, (GTAT)*n*, (ATTT)*n*, and (TTTG)*n* [42,86,89,90]. (GATA)*n* sequences, which to date have not been mapped in rice, are the most common tetra-nucleotide repeat, although only 270 of these motifs are found in the entire rice genome [91]. However, it should be kept in mind that it is the degree of polymorphism shown by microsatellites, rather than their abundance within the genome, that ultimately determines their usefulness in genomic analyses [92].

The motif (CGG)*n* has been reported to be very abundant in rice and is interspersed throughout the genome [89]. Several studies based on GenBank searches of rice sequences identified a variety of microsatellites motifs [83,86,90]. In a study by Panaud *et al.* [85], 34-bp oligonucleotides (representing two di-nucleotide, seven tri-nucleotide, and four tetra-nucleotide motifs) were synthesized and used as probes for library screening. Screening of a 15-kb insert genomic library suggested that the relative frequency of the various microsatellite motifs was inversely correlated with the size of the repeat motif [38]. This finding was consistent with previous reports based on GenBank searches in a range of plant species [39]. The microsatellite (GATA)*n* has also been frequently used for DNA fingerprinting. For example, Davierwala *et al.* [92] identified three polymorphic (GATA)*n*-harboring loci (OS1A6, OS1H10 and OS2E7) that contained 7–13 repeat motifs by probing a genomic library from the cultivated rice strain *Oryza sativa* var. Basmati-370 with a oligonucleotide (GATA)4 probe.

**Figure 2:**
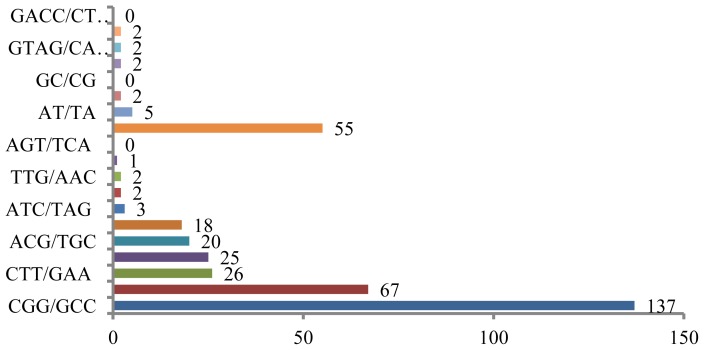
Frequencies of microsatellites in the rice sequences registered in the database [90].

Microsatellites can also be screened for using published rice sequences in the DDBJ databank, as numerous sequencing datasets, including those for EST sites, are available in this database. Homology searches can be performed to identify all di- and tri-nucleotide motifs, as well as four types of tetra-nucleotide repeats, and several of these are shown in Figure 2. Out of 11,798 total sequences, 369 contained complete repeats, and most types of repeat sequences, with the exception of (GC/CG)*n*, (AGT/TCA)*n* and (GACC/CTGG)*n*, were found. Sequences containing (CGG/GCC)*n* were most frequently identified in the published rice sequences, followed by sequences containing (GAG/CTC)*n* (Figure 2). Poly (CGG) loci were also abundant and were found throughout the rice genome [89], and the results presented here are likely representative of this feature of the rice genome. However, only a limited number of published rice sequences contain (AT/TA) sequences, although this motif is generally abundant in other plant genomes [83,84].

## 9. Microsatellites for Tracking Blast Resistance in Rice

Many *Pi* genes confer resistance to overlapping spectra of blast pathotypes, and it is often difficult to monitor for the presence of individual resistance genes and pyramid these in breeding lines using traditional phenotypic screening. Therefore, DNA markers provide a straightforward and rapid means to select for multiple blast resistance genes without performing extensive progeny testing or disease screening. DNA markers linked to several of the *Pi* genes have been localized on rice chromosomes, as well as markers for *Pi-ta* [93–95] and *Pi-b* [96]. Unfortunately, the majority of DNA markers for blast resistance are RFLPs, which are relatively labor intensive to analyze for use in breeding programs. Markers that can be analyzed by PCR are more amenable for breeding purposes, such as the ones developed for *Pi-2* [94] and *Pi-ta* [93].

The rice blast resistance gene *Pi-z*, which is present in the rice genotypes Zenith and Fukunishiki, represents a potential source of blast resistance for the northwestern Himalayan region of India. Sharma *et al.* [97] and Rathour *et al.* [98] both tested the reliability of microsatellite markers linked to *Pi-z* for assessing the blast resistance phenotype in commercially important crosses. A new set of microsatellite markers linked to *Pi-z* was developed by exploiting publicly available marker and genomic resources in rice. Of the three previously reported markers for *Pi-z*, only MRG5836 was found to be suitable for MAS. Among 17 microsatellites selected from the putative *Pi-z* locus, two (RM8225 and RM8226) co-segregated with MRG5836, and they were located at distance of 1.2–4.5 cM from the gene. In addition, a new microsatellite marker, SSR236, was derived from the (CT)16 repeat within the PAC clone P0502B12, and it showed even closer linkage to *Pi-z*. A survey of the allelic diversity at the loci containing the *Pi-z*-linked microsatellite markers revealed that the Fukunishiki- and Zenith-type alleles were not present in a majority of the local *indica* rice genotypes. Therefore, as these markers are polymorphic between the *Pi-*z donors and the great majority of local *indica* rice strains that were tested, they can be used as selection tools in rice breeding programs aimed at improving blast resistance in local rice varieties [99]. Conaway-Bormans *et al.* [63] identified three microsatellite markers that mapped at a distance of 0.0–11.5 cM from *Pi-z* in several different crosses segregating for the gene. Genetic distances between markers often vary in different mapping populations due to differences in the genetic backgrounds of the parental genotypes [100]. The gene *Pi-z* has been reported to be allelic with, or at least closely linked to, three other blast resistance genes, *Pi-2*, *Pi-zt* and *Pi-9*, which map close to the centromere of chromosome 6 [8,101]. Furthermore, the SSR236 marker has now bridged the 7.5-cM gap between the microsatellite markers RM8226 (54.1 cM) and RM3330 (61.6 cM) in the current version of the IRMI SSR map [99]. In addition, RM208 has been linked to *Pi-b* resistance, YL155 and YL183 have been linked to *Pi-ta* resistance in *indica*, and AP5659-1 has been linked to *Pi-z* resistance [65,73,102].

The *Pi20(t)* gene was identified from 160 Chinese *Magnaporthe oryzae* isolates, and among these, isolate 98095 can specifically differentiate the *Pi20(t)* gene present in cv. IR24. Two flanking and three co-segregating microsatellite markers for *Pi20(t)*, which is located near the centromeric region of chromosome 12, were identified using 526 highly susceptible F_2_ plants derived from a cross between Asominori (highly susceptible) and IR24 (resistant). The microsatellite OSR32 was mapped at a distance of 0.2 cM from *Pi20(t)*, and the microsatellite RM28050 was mapped to the other side of *Pi20(t)* at a distance of 0.4 cM. The other three microsatellite markers, RM1337, RM5364 and RM7102, were observed to co-segregate with *Pi20(t)*. In particular, RM1337 and RM5364 were found to be reliable markers of the resistance mediated by *Pi20(t)* in a wide range of elite rice germplasms from China. Therefore, these markers are useful tags for use in marker-assisted rice breeding programs aimed at incorporating *Pi20(t)* into advanced rice breeding lines [123]. Sharma *et al.* [127] concluded that RM25 and RM310 are two microsatellite markers linked to blast resistance in the Laxmi cultivar. These two markers are located 4.5 cM apart on chromosome 8 in rice [42]. An advanced backcross population of BC_3_F_3_ lines derived from the rice varieties Vandana and Moroberekan was analyzed for blast resistance by Wu *et al.* [128], and they identified four microsatellite markers (RM21, RM168, RM215 and RM250) that were significantly associated with the resistance gene.

This locus was mapped by Liu *et al.* [119] to a 5.8-cM interval bounded by RM5647 and RM8018 on the short arm of chromosome 8. This novel resistance gene has been tentatively designated as *Pi36(t).* According to a report by Liu, *Pi-1* is located 6.8 cM away from the RM144 microsatellite; their results indicate that the physical distance between these two loci was between 57 and 72 kb [129]. Chen *et al.* [114] determined that the genetic distance between *Pi-1* and the MRG4766 microsatellite marker was 1.3 cM. More recently, there has been a report concerning the identification of rice blast resistance using RM144 [130], indicating that it may be possible to identify this resistance gene using microsatellite markers. The known blast resistance genes and their linked microsatellite markers are shown in Table 7.

The Korean cultivar Suweon 365 carries three major resistance genes, *Pi18*, *Pi21(t)* and *Pi22(t)*, that are effective against the Korean isolates KI-313, KJ-101, and KJ-201, respectively, and these genes have been tracked using microsatellite markers in an F_2_ population derived from a cross of the Suweon and Chucheongbyeo cultivars [131]. Microsatellite markers were used to map the gene *Pi-k*^h^, which confers resistance to blast races in the Himalayan region of Northeast India [121]. Pinheiro *et al*. [13] developed the cultivar Cica-8 by crossing the susceptible cultivar Metica-1 to the resistant cultivar Cica-8, and they found that one microsatellite marker, RM7102, was closely linked to the resistant allele. Yang *et al.* [132], while working on identifying and mapping the *Pi41* gene, utilized resistant cultivar 93-11 and susceptible cultivar Nipponbare, as well as an F_2_ population derived from a cross of these two cultivars. They studied 180 microsatellite markers and identified seven markers that were linked to *Pi41*, one of which was RM7102. The markers RM144, RM224 and yca72 are linked to the resistance genes *Pi-k*^s^, *Pi1* and *Pia*, respectively [133,134]. Koide *et al.* [135] identified four microsatellite markers, RM7419, RM1268, RM6648 and RM5811 that were linked to *Pish*. With respect to *Pib*, both a gene-specific marker, *Pibdom*, and a co-segregation marker, RM208, have been reported [73]. Gouda *et al.* [136] found that the two microsatellite markers RM5926 and AP5659-5 were tightly linked to the *Pi-1* and *Piz-5* genes, respectively, in PRR78. Pan *et al.* reported that *Pii(t)*, *Pi3(t)*, *Pi5(t)* and *Pi15(t)* are all located within the same interval on chromosome 9 in rice [124]. Moumeni and Leung reported that three microsatellite markers, RM224, RM179 and RM 277, on chromosomes 11 and 12 were tightly linked to components of rice blast resistance [137]. In addition, Liu *et al.* [109] revealed that RM247 and RM463 were located on chromosome 12 and were linked to the *Pi39* resistance gene. Abedi *et al.* [138] also identified four microsatellite markers, RM224, RM277, RM463 and RM179, which are linked to resistance genes on rice chromosomes in Iranian rice genotypes. This suggests there exists at least one race-specific resistance gene among the genetic sources of genotypes that confers resistance functions to the blast races. The marker AP5930, which is linked to *Piz-5* [65], and RM206, which is linked to *Pi54* [121], were used for foreground selection in both backcrossed and “selfed” generations. Finally, Singh *et al.* [139] also used a microsatellite marker (RM6100) when incorporating blast resistance into PRR78, an elite Basmati rice restorer line, through marker-assisted backcross breeding.

A large number of databases are available for selecting molecular markers linked to the *Pi* genes (http://www.gramene.org). Most of the known blast resistance genes have been mapped to chromosomes 12, 11 and 6 [140–143] through the work of a large number of researchers. For example, *Pi-1(t), Pi-2(t)* and *Pi-4(t)* were mapped to chromosomes 11, 6 and 12, respectively [144]. The microsatellite markers RM168, RM8225, RM1233, RM6836, RM5961 and RM413 were analyzed by Ashkani *et al*. [145], and they were linked to blast resistance genes specific to pathotype P7.2. Furthermore, molecular marker-assisted rice breeding programs have been developed with the aim of developing durable blast resistance in rice cultivars by pyramiding the resistance genes *Pi-1(t)*, *Pi-2(t)* and *Pi-33(t)*, which shows potential for controlling blast pathogen populations in Latin America [146].

## 10. Microsatellite Markers and Marker-Assisted Selection

The continued development of molecular markers promises to overcome most of the existing limitations associated with morphological markers. In particular, the tight linkage of a molecular marker to a gene can be exploited for indirect selection of traits in a breeding program, which is also referred to as MAS. Advances in the development of molecular markers and their implementation in cereal breeding programs have led to a greater understanding of rice genetics and genomes. Disease assays to evaluate resistance to rice blast are time-consuming and laborious procedures that require specialized facilities. However, PCR-based analyses promise to significantly reduce the amount of labor needed for evaluating phenotypes by prescreening with MAS. To accelerate the effectiveness of MAS, the map locations of target QTL must first be precisely determined, and several flanking markers must be developed [147]. Indeed, four QTLs derived from upland rice cultivars that control partial resistance to rice blast have been successfully pyramidized into lowland rice cultivars using MAS [147]. However, the successful application of MAS requires extremely tight linkages between markers and phenotypic traits. Some successful examples of using microsatellite markers in MAS to introduce blast resistance in rice are shown in Table 8.

Compared with previously reported RFLP markers linked to the *Pi-1(t)* gene [94,155], microsatellite markers are potentially more useful in developing countries where financial support is the principal limiting factor to establishing MAS rice breeding programs. Fuentes *et al.* [16] discussed the utility of DNA markers in MAS and gene pyramiding in rice breeding programs aimed at improving blast resistance. Indeed, PCR-based allele-specific markers provide an efficient system for MAS in blast resistance breeding programs [122]. However, the importance of using DNA markers within or flanking genes of interest during MAS strategies in rice should be stressed [73]. Finally, it is possible that several blast resistance genes could be combined using MAS in a single genetic background to develop rice cultivars with broad-spectrum durable resistance to blast [156].

## 11. Future Directions of Microsatellite Marker Research

Microsatellite markers provide an invaluable tool for plant geneticists and breeders, as detecting polymorphisms are a limiting factor in many breeding strategies. In the long term, the development of allele-specific markers for genes controlling disease resistance traits (e.g., blast disease resistance) will become increasingly important in the science of rice breeding. The choice of the most appropriate marker systems for a given program must be made on a case by case basis and will depend on many issues, including the availability of technology platforms, costs for marker development, species transferability, information content and ease of documentation. In addition, a higher degree of genetic variability and the localization of more markers on the rice linkage map will provide additional resources for genomic analysis and rice breeding. Therefore, there exist great opportunities for more efficient breeding programs and faster development times for new rice varieties resistant to biotic diseases in the future.

## 12. Conclusions

Molecular mapping of rice populations is a prerequisite to identifying markers closely linked to the desirable *Pi* resistance gene. In particular, microsatellite markers have become very important in rice breeding. Although many marker types exist within the rice genome, microsatellite markers are used in a wide range of studies due to their small size and repetitive nature, and they have played an important role in the identification of numerous important genetic loci. Microsatellite markers are also widely used in MAS programs to develop durably resistant cultivars against specific diseases. In recent years, the popularity of microsatellite-based markers has increased considerably. Microsatellites have been found to be highly polymorphic, genome-specific, abundant and co-dominant, and they have become important genetic markers in rice breeding programs for improving blast resistance.

This review has been specifically written for readers who want to use microsatellite markers for blast resistance improvement programs in rice cultivars, and will likely be useful for studies aimed at identifying the linkages between blast resistance genes and microsatellite markers. The techniques described will likely also prove useful for marker-assisted selection in the absence of appropriate pathogen isolates or when funds are limited, as is the case in most developing countries. Investigating the abundance and structure of rice microsatellite repeats, as well as their allelic variations and distributions should extend our knowledge concerning this class of tandem repeat in the rice genome. We hope that some of the ideas proposed in this article will encourage the rice scientific community to work together to convert rice from a model crop species into a model species for marker-assisted breeding.

## Figures and Tables

**Table 1:** Important feature of different types of molecular markers.

S.N.	Feature	RFLP	RAPD	AFLP	SSRs	SNPs
1	DNA Require (μg)	10	0.02	0.5–1.0	0.05	0.05
2	PCR based	No	Yes	Yes	Yes	Yes
3	DNA quality	High	High	Moderate	Moderate	High
4	No. of Polymorph loci analyzed	1–3	1.5–50	20–100	1–3	1
5	Type of polymorphism	Single base change, insertion, deletion	Single base change, insertion, deletion	Single base change, insertion, deletion	Change in repeat length	Single nucleotide change, insertion, deletion
6	Dominance	Co-dominant	Dominant	Dominant/Co-dominant	Co-dominant	Co-dominant
7	Reproducibility	High	Unreliable	High	High	High
8	Ease of use and development	Not easy	Easy	Easy	Easy	Easy
9	Automation	Low	Moderate	Moderate	High	High
10	Cost per analysis	High	Low	Moderate	Low	Low
11	Developmental cost	Low	Low	Moderate	High	High
12	Need for sequence data	Yes	No	No	Yes	Yes
13	Accuracy	Very high	Very low	Medium	High	Very high
14	Radioactive detection	Usually yes	No	No	No	Yes
15	Genomic abundance	High	Very high	Very high	Medium	Medium
16	Part of genome surveyed	Low copy coding regions	Whole genome	Whole genome	Whole genome	Whole genome
17	Level of polymorphism a	Low	Low to moderate	Low to moderate	High	High
18	Effective multiplex ratio b	Low	Medium	High	Medium	Medium
19	Marker index c	Low	Medium	High	Medium	Medium
20	Inheritance	Codominant	Dominant	Dominant	Codominant	Codominant
21	Detection of alleles	Yes	No	No	Yes	Yes
22	Utility for genetic mapping	Species specific	Cross specific	Cross specific	Species specific	Species specific
23	Utility in Marker assisted selection	Moderate	Low to moderate	Low to moderate	High	Low to moderate
24	Cost and labour involved in generation	High	Low-moderate	Low-moderate	High	High

a Level of polymorphism (average heterozygosity) is an average of the probability that two alleles taken at random can be distinguished;

b Effective multiplex ratio is the number of polymorphic loci analysed per experiment in the germplasm tested;

c Marker index is the product of the average expected heterozygosity and the effective multiplex ratio.

Source: [29–33].

**Table 2:** Classification of microsatellites.

(**A**) Based on the arrangement of nucleotides in the repeat motifs [34–36]

Pure or perfect or simple perfect (CA)*n* Simple imperfect (AAC)*n* ACT (AAC)*n* + 1
Compound or simple compound (CA)*n* (GA)*n*
Interrupted or imperfect or compound imperfect (CCA)*n* TT (CGA)*n* + 1

(**B**) Based on the number of nucleotides per repeat [31]

Mononucleotide (A)*n*
Dinucleotide (CA)*n*
Trinucleotide (CGT)*n*
Tetranucleotide (CAGA)*n*
Pentanucleotide (AAATT)*n*
Hexanucleotide (CTTTAA)*n* (*n* = number of variables)

(**C**) Based on location of SSRs in the genome [31]

Nuclear (nuSSRs)
Chloroplastic (cpSSRs)
Mitochondrial (mtSSRs)

**Table 3:** Some potential benefits and weakness of the most commonly used markers.

Markers type	Benefits	Weakness
RFLP	-Co-dominant -Genomic abundance high -Highly reproducible -Better genome exposure -Applicable across the species -No need for sequence information -Reliably used in plants	-Need high-quality DNA -Laborious (compared to RAPD) -Complex to automate -Radioactive labeling essential -Characterization of probe is essential
RAPD	-Genomic abundance high -Better genome coverage -Sequence information unneeded -Perfect for automation -Requires less DNA -No radioactive labeling -More rapid	-No need of probe information -Dominant markers -Not reproducible -Not suitable for across species -Not well tested
SSR	-Easy to automate -Genomic abundance high -Highly reproducible -High polymorphism -Multiple alleles -Moderately genome coverage -No radioactive labeling	-Not well-examined -Cannot suitable across species -Sequence information needed
AFLP	-High polymorphism -Genomic abundance high -Can be used across species -No need for sequence information -Useful in preparing counting maps -Works with smaller RFLP fragments	-Very tricky due to changes in materials used -Not reproducible -Very good primers needed
Sequence-tagged site (STS)	-Helpful in preparing counting maps -Highly reproducible -No radioactive labeling -Can use filters many times -Moderate genome coverage	-Need sequence information -Out of the target sites, mutation detection not possible -Laborious -Cloning and probe characterization required
Minisatellites	-Highly polymorphic -Multiallelic markers -High reproducibility -Low cost	-Many informative bands per reaction -Band profiles can not be interpreted in terms of loci and alleles

Source: [29].

**Table 4:** A comparison of the main features of microsatellite-based markers.

Features	Marker type		
	Microsatellite	ISSR	SAMPL
Abundance	High	High	Medium/high
Locus specifity	Yes	No	No
Nature of polymorphism	Variation in repeat length/number of motifs	Base changes (insertions, deletions), variation in microsatellite repeat length/number of motifs	(insertions, deletions) variation in SSR repeat length/number of motifs
Level of Polymorphism	High/very high	High/medium	High
Inheritance mode	Codominance	Dominance/codominance	Codominance/dominance
Reproducibility	High	High/medium	High
Sequence information required	Yes	No	No
Technical demands	medium/low (except for library construction and screening)	low/medium	medium
Costs	Medium	Low	Medium
Labor	High (a labor-consuming step of library construction and screening)	Low	Medium
Time	Usually a time-consuming step of library construction and screening is needed	Low	Medium
Main applications	Linkage mapping, studies on genetic diversity, gene tagging	Identification of cultivars, phylogenetic studies	Studies on genetic diversity, linkage Mapping
Main advantages	High level of polymorphisms (up to 26 alleles), co-dominant mode of inheritance, very high reproducibility	Multilocus and highly polymorphic pattern production per reaction, technical simplicity, low expenses	Amplification of many informative bands per reaction, high reproducibility
Problems	Frequently a small number of potential microsatellite loci are identified, polymerase slippage when analysing mono- and di-ucleotide repeats, co-migrating fragments not always are homologous	Band profiles cannot be interpreted in terms of loci and alleles, dominance of alleles (frequently), similar-sized fragments may not be homologous	Relatively time consuming and labor-intensive procedure, high complexity of amplification profiles may occur

Source: [57].

**Table 5:** Abundance of DNA markers discovered and developed in rice.

Crop	Genome size (MB)	RFLP	RAPD	AFLP	SSR	SNP
Rice	415–460 b	3,553 b	133 b	1,062 b	12,992 b	5,418,373 a

a www.ncbi.nlm.nih.gov;

b Gramene web browser (http://www.gramene.org).

Source: [82].

**Table 6:** Recommended websites for microsatellite markers.

**Gramene web browser** http://www.gramene.org	Gramene is a data resource for comparative genome analysis in the grasses, in particular the cereals: rice, maize, oats *etc.* It provides comprehensive and in-depth information regarding markers used for mapping plant species such as RAPD, SSR, AFLP and RFLP.
**MSU rice genome annotation projct** http://rice.plantbiology.msu.edu	This website provides genome sequence from the Nipponbare subspecies of rice and annotation of the 12 rice chromosomes.

Source: [82].

**Table 7:** Microsatellite markers linked to rice blast disease resistance gene.

Gene name	Chromosome	Linked microsatellite marker	Rice variety	References
*Pi-2(t)*		RM140	Recombinant inbred lines	[103]
*Pi33*	8	RM72, RM44	IR64 × Azucena and Azucena × Bala	[104]
*Pi-1(t)*	11	RM1233*I and RM224	Near-isogenic lines C101LAC and C101A5	[16]
*Pi-k^h^*	11	RM1233*I and RM206	Near-isogenic lines C101LAC and C101A5	[73]
*Pi-k^s^*	11	RM224	Near-isogenic lines C101LAC and C101A5	[73]
*Pi37*	1	RM140, RM302, RM212, FPSM1, FPSM2, FPSM4	-	[105]
*Pi-b*	2	RM166, RM138, RM208, RM266, RM138	Tohoku IL9 and Sasanishiki	[74]
*Piz-t*	6	RM225, RM226	Isogenic line C101A51 and cultivar CO39	[106]
*Pi9*	6	RM136	Cultivar TP309	[107]
*Pid2*	8	RM263	Variety LTH and Digu	[108]
*Pi36*	8	RM544	Q15 and Tsuyuake	[109]
*Pita*	12	OSM89, RM155, RM7102	Yashiro-mochi and Tsuyuake	[110]
*Pi27(t)*	1	RM151, RN259	Q14 and Q61	[111]
*Pitp(t)*	1	RM246	CO39 and Tetep	[112]
*Pi35(t)*	1	RM1216, RM1003	Hokkai 188 and Danghang-Shali	[113]
*Pi37*	1	RM302, RM212, FPSM1, FPSM2, FPSM4	C101PKT, CO39 and AS20-1 crossed with cultivar St. No. 1	[114]
*Pid1(t)*	2	RM262, RM208	Lijiangxintuanheigu (LTH) and Jiangnanxiangnuo (JNXN) crossed with Digu	[115]
*Pig(t)*	2	RM166	Q61 and Q14	[111]
*Piy1*	2	RM3248, RM20	Lijiangxintuanheigu (LTH) and Yanxian No.1	[116]
*Piy2*	2	RM3248, RM20	Lijiangxintuanheigu (LTH) and Yanxian No.1	[116]
*Pi39*	4	RM5473, RM3843	Mineasahi and Chubu 111	[117]
*Pi40(t)*	6	RM527, RM3330	Co39 and IR50 cross with IR65482-4-136-2-2	[118]
*Pi36*	8	RM5647	Aichi Asahi and Lijiangxintuanheigu (LTH) crossed with Q61	[119]
*Pi38*	11	RM206, RM21	CO39 and Tadukan	[120]
*Pik-h*	11	RM224, RM144, RM1233, RM144, RM1233, RM224, RM206, TRS33, TRS26, RM144	HP2216 and Tetep	[73,121]
*Pik-s*	11	RM1233, RM224, RM144, RM1233, RM224, RM144	-	[73]
*Pita-2*	12	OSM89, RM155, OSM89, RM7102, OSM89, RM712	Koshihikari cross with Fukunishiki (Piz+), Toride 1 (Piz-t+), K59 (Pit+), Kanto 51 (Pik+), Tsuyuake (Pik-m+), K60 (Pik-p+), BL 1 (Pib+), Yashiromochi (Pita+), and Pi No.4 (Pita-2+)	[122]
*Pi20(t)*	12	RM1337, RM7102, RM54	Asominori and IR24	[123]
*Pi15*	9	RM316	Q61 and GA25	[124]
*Pi36*	8	RM5647-CRG2	Aichi Asahi and Lijiangxintuanheigu (LTH) cross with Q61	[119]
*Pi37*	1	RM543-FPSM1	cvs. C101PKT, CO39 and AS20-1 crossed with cultivar St. No. 1	[114]
*Pi39*	12	RM27933-RM27940	Tsuyuake crossed with Q15	[109]
*-*	4	RM 5757	White Ponni × Moroberekan	[125]
*-*	4	RM 451	White Ponni × Moroberekan	[125]
*-*	2	RM 492	White Ponni × Moroberekan	[125]
*-*	2	RM208	Gulfmont*2/Te-Qing F12, Maybelle*2/Te-Qing F2	[73]
*Pi-k*^s^	11	RM224	Maybelle*2/Kaybonnet F2, Maybelle*2/Lemont F2, Maybelle*2/Bengal F2, Maybelle*2/M-201 F2	[73]
*-*	2	Pibdom	Gulfmont*2/Te-Qing F12	[73]
*-*	12	RM155	Maybelle*2/Kaybonnet F2	[73]
*-*	12	RM7102	Kaybonnet/M-204 F2	[73]
*Pi40*	6		wild Oryza species (*O. australiensis*)	[118]

Source: [126].

**Table 8:** Examples of MAS application for blast resistance in rice.

Application	Traits	Gene/QTLs	Markers used	References
Gene surveys in parental material	Blast disease	*Pi-z*	Microsatellite	[65]
Gene surveys in parental material	Blast disease	*Pi-ta*	Gene-specific marker	[148]
MAS applied for backcross breeding	Blast	*Pi1*	Microsatellite and ISSR	[149]
Marker assisted backcrossing	Submergence tolerance, blast disease resistance, quality	*Subchr9* QTL, *Xa21*, *Bph* and blast QTLs, and quality loci	Microsatellite and STS	[150]
Marker assisted backcrossing	Blast disease	-	Microsatellite	[151]
MAS applied for backcross breeding	Blast resistance BB	*Pi1* and *Pi2* for blast resistance	Microsatellite	[152]

Source: [153,154].
